# The Current State of Visualization Techniques in Endoscopic Skull Base Surgery

**DOI:** 10.3390/brainsci12101337

**Published:** 2022-10-03

**Authors:** Jakub Jarmula, Erion Junior de Andrade, Varun R. Kshettry, Pablo F. Recinos

**Affiliations:** 1 Department of Neurological Surgery, Cleveland Clinic Lerner College of Medicine, Case Western Reserve University, Cleveland, OH 44106, USA; 2 Section of Skull Base Surgery, Rose Ella Burkhardt Brain Tumor and Neuro-Oncology Center, Neurological Institute, Cleveland Clinic, Cleveland, OH 44195, USA; 3 Section of Rhinology, Sinus & Skull Base Surgery, Head and Neck Institute, Cleveland Clinic, Cleveland, OH 44195, USA

**Keywords:** skull base surgery, neuro-oncology, neuronavigation, neuroendoscopy, ultrasonography

## Abstract

Skull base surgery has undergone significant progress following key technological developments. From early candle-lit devices to the modern endoscope, refinements in visualization techniques have made endoscopic skull base surgery (ESBS) a standard practice for treating a variety of conditions. The endoscope has also been integrated with other technologies to enhance visualization, including fluorescence agents, intraoperative neuronavigation with augmented reality, and the exoscope. Endoscopic approaches have allowed neurosurgeons to reevaluate skull base neuroanatomy from new perspectives. These advances now serve as the foundation for future developments in ESBS. In this narrative review, we discuss the history and development of ESBS, current visualization techniques, and future innovations.

## 1. Introduction

Over the last century, the evolution of skull base surgery has, in large part, been dependent on technology to improve our ability to visualize and work in small, deep, and dark corridors. Starting only with the naked eye and candlelight, neurosurgery progressed through improving forms of visualization and illumination to surgical loupes and headlights, to microscopes, endoscopes, and exoscopes. Improved magnification and illumination have allowed neurosurgeons to move from very large open craniotomy approaches to smaller, more tailored open and keyhole approaches, and to endonasal approaches, which use the natural nasal cavity to access the skull base [1].

Endoscopic skull base surgery (ESBS) in particular has emerged as an established surgical approach for specific pathologies, including pituitary macroadenoma, cerebrospinal fluid rhinorrhea, craniopharyngioma, midline chordoma, and anterior skull base meningioma. Beyond neurosurgery, the use of ESBS has grown in otolaryngology, where ESBS is an established approach for treating olfactory neuroblastoma (in particular, Kadish A and B tumors), juvenile nasopharyngeal angiofibroma, cholesterol granuloma, and recurrent nasopharyngeal cancer. ESBS is also an option for select patients with vasomotor rhinitis, allergic rhinitis, and sinonasal squamous cell carcinoma [2]. Although adoption rates vary by pathology, the past 30 years have seen an increasing trend in the use of ESBS, with a majority (53.7%) of nonfunctioning pituitary adenomas treated with ESBS compared to non-endoscopic approaches [3]. One survey found ESBS was used by 80% of skull base surgery respondents, comprising both neurosurgeons and otolaryngologists [4].

Compared to traditional microscopic approaches, ESBS offers the advantages of improved magnification and illuminance of structures, panoramic visualization with angled lenses, and maneuverability around important neurovascular structures (e.g., internal carotid arteries, cranial nerves) [5]. ESBS offers the advantages of improved neurological function (e.g., greater visual field improvement for pituitary macroadenoma, greater hypothalamic preservation for craniopharyngioma) and postoperative outcomes (e.g., shorter hospital length of stay, reduced patient discomfort) relative to open microsurgical approaches [6]. However, ESBS has notable limitations. It provides a two-dimensional (2D) monocular view, requires two co-surgeons, and has certain anatomic constraints—offering the best outcomes for smaller, midline pathologies—compared to microscopic surgery. Complications in ESBS include cerebrospinal fluid rhinorrhea (8.9% incidence), vascular injury and endocrine dysfunction (2.0%), central nervous system infection (1.7%), and death (0.4%) [7]. ESBS represents an additional approach for treating skull base pathologies and requires judicious use by the skull base surgeon to offer patients the best individualized treatment plan.

In this contemporary narrative review, we provide a comprehensive description of the current state of visualization techniques in ESBS. We present a selection of cited articles based on a search of MEDLINE/PubMed for articles published within the last ten years and foundational articles determined by the authors to be relevant to this topic. We discuss the history and development of ESBS, current visualization techniques, and future innovations.

## 2. The History and Development of Endoscopic Skull Base Surgery

**Figure 1:**
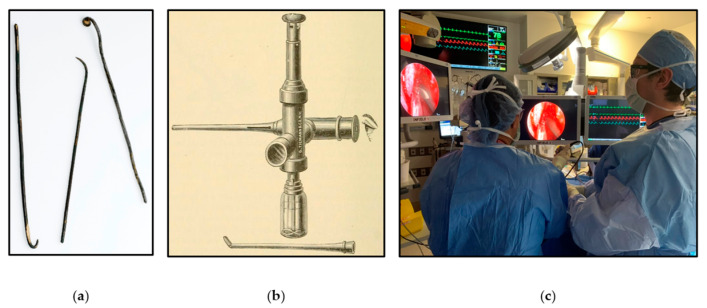
(**a**) Examples of instruments used for excerebration in Ancient Egypt. Reprinted/adapted with permission from Ref. [12]. 2022, Wellcome Collection. (**b**) Lamp-lit endoscope designed by Desormeaux. Reprinted/adapted with permission from Ref. [12]. 1891, Lea Brothers & Company. (**c**) Current endoscopic endonasal operating room suite at the Cleveland Clinic. The high-definition surgical endoscope setup improves visualization and ergonomics for two simultaneous surgeons. Reprinted/adapted with permission from Ref. [12]. 2022, Pablo F. Recinos, M.D.

The ancient Egyptians conducted the first recorded transnasal procedures during mummification, where long instruments were used to perform excerebration (i.e., the removal of the brain and its surrounding structures) through the nose [8] (Figure 1). The concept behind endoscopy originated with Phillip Bozzini, who invented the *lichtleiter* (“light conductor”) in 1805 [9]. Bozzini used candlelight and a small mirror to allow internal visualization through natural orifices or small incisions. In 1853, Antonin Jean Desormeaux coined the term *l’endoscope* to describe his oil lamp-lit instrument for visualizing the internal genitourinary system (Figure 1). However, endoscopic illuminance was dependent on candles and oil lamps until the invention of the incandescent lightbulb by Thomas Edison in 1879. In 1906, Hermann Schloffer performed the first transnasal neurosurgical procedure, guiding past the sphenoid bone to resect a pituitary tumor—termed the transsphenoidal approach. The development of fiberoptics in the 1950s by physicists Harold Hopkins and Narinder Singh Kapany and the rod-lens system by Harold Hopkins in the 1959 led to the modern fiberoptic endoscope [10]. In 1963, Gerard Guiot reported the first fiberoptic endoscopic transnasal transsphenoidal approach [11]. However, rapid advancements made the operating microscope the gold standard for transsphenoidal and transcranial approaches to skull base lesions during this time period [9].

The development of coupled-charge devices by Bell Laboratories in 1969 gave rise to video-based endoscopy, which was further refined by Karl Storz during the second half of the twentieth century. In 1992, the first endoscopic endonasal pituitary resection was reported by Roger Jankowski et al. [13], and in 1997, Ricardo Carrau and Hae-Dong Jho published the first large case series of fully endoscopic endonasal pituitary surgery in 50 patients [14]. Subsequent refinements in visualization technology, instrumentation, anatomical knowledge, and reconstruction techniques have made ESBS a widely adopted surgical practice today [15] (Figure 1).

## 3. The Current State of Visualization Techniques in Endoscopic Skull Base Surgery

### 3.1. Illuminance and Brightness

**Figure 2:**
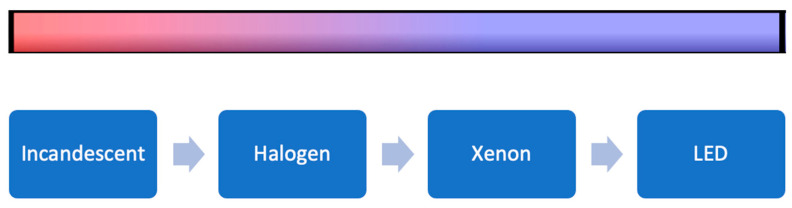
Light sources from high to low heat production. Incandescent lightbulbs produce more heat (red) than LED light sources (blue). LED: light emitting diode. Reprinted/adapted with permission from Ref. [18]. 2022, Erion Junior de Andrade, M.D., M.Sc.

Endoscopic image quality depends on specific image characteristics, including illuminance, brightness, resolution, and dimensionality. Illuminance is the amount of light distributed across a surface area [16]. Proper illuminance of anatomic structures is critical for ESBS. A variety of light sources have been developed with different emission spectra. Currently, most endoscopes use xenon light sources that produce excellent illuminance with their high content of blue wavelength light to provide accurate color representation of tissues. However, these light sources carry the risk of thermal tissue damage, especially with high-watt lightbulbs. Light-emitting diode (LED) light sources have also been developed. These instruments carry a lower risk of thermal tissue injury while providing adequate light quality for ESBS [17] (Figure 2).

Projecting sufficient light towards the structure of interest (i.e., illuminance) is equally as important as capturing enough returning light to form a recognizable image (i.e., brightness). Brightness is the amount of illuminance perceived by an observer [16]. In ESBS, brightness relates to the amount of light radiating from the visualized structures towards the endoscope light sensor, which is then transmitted to the monitor and seen by the surgeon. Brightness is directly proportional to illuminance and inversely proportional to distance squared. Appropriate brightness can be ensured by increasing the endoscope light source illuminance or decreasing the distance between the structure of interest and the endoscope. However, maximal brightness cannot always be ensured. For example, decreasing illuminance or increasing working distance to reduce thermal tissue injury can limit image brightness.

### 3.2. Resolution

In addition to brightness, resolution is important for generating precise images during surgery. Currently, endoscopes are available in high definition (HD) (1280 × 720 pixels), full HD (1920 × 1080 pixels), and ultra-HD/4K resolution (3840 × 2160 pixels) with the aim of enhancing the visualization of anatomic structures [19]. Additional resolution could be valuable when evaluating detailed anatomy, such as the tumor-tissue interface or intradural features. In response, features such as digital zoom enhance visibility at a fixed distance while maintaining adequate resolution. Digital zoom can improve instrument maneuverability in small spaces, such as the nasal cavity, by allowing enhanced visibility at a longer distance so as other surgical instruments do not collide with the endoscope rod. However, unlike optical zoom, digital zoom reduces the field of view.

**Figure 3:**
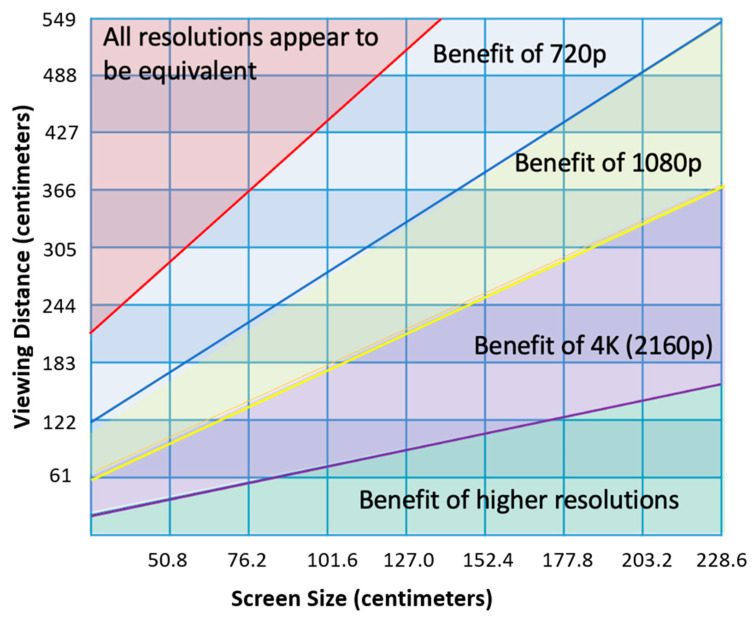
Graph of the ability of the human eye to perceived screen resolution based on screen size (*x*-axis) and distance from monitor (*y*-axis). Reprinted/adapted with permission from Ref. [21]. 2022, Carlton Bale, M.S., M.B.A.

It is important to appreciate the effects of viewing distance and screen size on perceived resolution by the surgeon. The recommended viewing distance between the surgeon and monitor ranges between 80–120 cm [20]. However, a typical viewing distance can range from 99–152 cm. With a standard 76.2-cm monitor, any improved resolution from an ultra-HD system can be perceived only when standing less than 122 cm away (Figure 3). With a standard viewing distance of 152 cm, any improved resolution from an ultra-HD system can only be perceived with a 101.6-cm or greater size monitor. Therefore, a larger viewing monitor is needed at standard working distances in order to perceive the advantages of a higher resolution system. However, larger monitors require additional physical space in the operating room, and high-resolution systems generate larger image and video files that result in a longer time to transfer these files to the medical chart or data storage device.

Beyond projecting images onto a two-dimensional monitor, certain endoscope systems have been developed to provide three-dimensional (3D) endoscopy [22]. Stereoscopic images generated by 3D endoscopy provide depth perception that facilitates surgery and is familiar to those accustomed to working stereoscopically with operating microscopes; however, certain users experience nausea, headache, or visual fatigue, limiting its adoption. Three-dimensional endoscopy presents a learning curve for users because of its differences from traditional endoscopy. In addition, the limitations of 2D images in traditional endoscopy can be overcome by the depth of field gained by seeing dynamic camera motion and by proprioceptive information gained from instruments moving in and out of the surgical corridor. Three-dimensional endoscopy remains a modern technology that requires more investigation to inform optimized settings for safe, effective ESBS.

### 3.3. Neuronavigation

**Figure 4:**
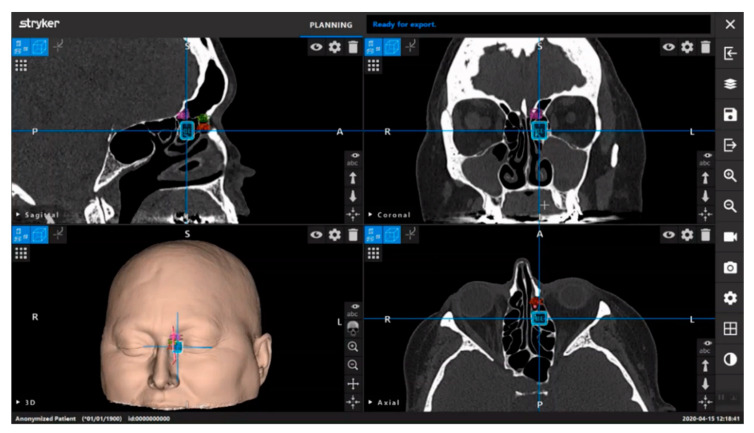
Preoperative planning with the Stryker Scopis neuronavigation system (Stryker Corporation, Kalamazoo, MI, USA). This system utilizes “building blocks” technology to highlight designated anatomical landmark. Reprinted/adapted with permission from Ref. [25]. 2022, Stryker Corporation.

Neuronavigation utilizes preoperative images and sensors to relate intraoperative coordinates to locations on imaging. Current systems can use optical image guidance, consisting of infrared light-detecting cameras and a reference array attached to the head holder, or electromagnetic tracking, which measures the electromagnetic field generated by a magnetic reference array typically placed on the patient’s forehead [23]. Neuronavigation systems undergo calibration and reference these markers to generate images with overlayed location coordinates. Surface contour matching requires identifying points on the patient’s surface with a designated probe to map the patient’s surface features. The computer-generated reconstruction of the patient is then referenced to map the current location of calibrated instruments in relation to preoperative imaging [24]. These systems utilize computer modeling to aid the surgeon pre- and intra- operatively (Figure 4). 

Outside of the operating room, adaptations of these systems into virtual reality platforms have been incorporation into neurosurgical training [26]. For ESBS, these platforms include the Dextroscope (Volume Interactions, Bracco Group, Milan, Italy) and NeuroTouch Endo (National Research Council of Canada, Ottawa, ON, Canada). These virtual reality platforms provide trainees with haptic feedback during part-task training and scenario simulations to promote operative skill development [27]. The advantages of virtual reality-based training platforms extend to their potential use in tracking trainee progress to provide feedback on trainee development and training program curricula.

**Figure 5:**
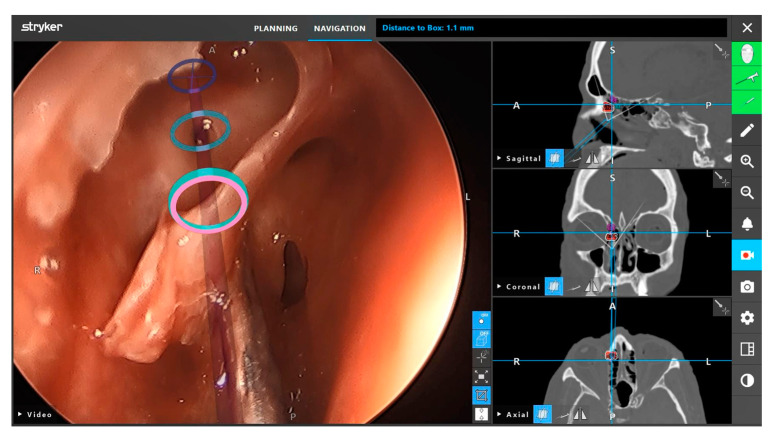
Augmented reality superimposed onto live endoscopic video with the Stryker Scopis navigation system (Stryker Corporation, Kalamazoo, MI, USA). Reprinted/adapted with permission from Ref. [25]. 2022, Stryker Corporation.

**Figure 6:**
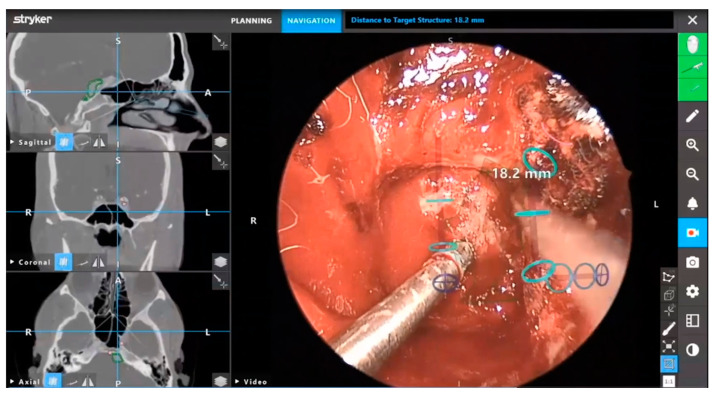
Augmented reality with live endoscopic video during ESBS to highlight the location of the basilar and internal carotid arteries. Augmented reality with the Stryker Scopis navigation system (Stryker Corporation, Kalamazoo, MI, USA). Reprinted/adapted with permission from Ref. [25]. 2022, Stryker Corporation.

Beyond virtual reality, the surgeon can outline key anatomical features on the screen that can be superimposed onto endoscopic imaging to provide augmented reality viewing (Figure 5 and Figure 6). First developed in the 1980s for operative microscopes, augmented reality provides personalized anatomical information. These systems can provide submillimeter accuracy that is integrated into one image, reducing the need for surgeons to view separate endoscopic and neuronavigation monitors [28]. Skull base anatomy presents many challenges, including anatomical variants that can affect surgical orientation. Augmented reality presents surgeons with an additional means of enhancing intraoperative orientation and preventing neurovascular injury [29]. Although current systems are focused on incorporating preoperative imaging, the use of intraoperative imaging can provide augmented reality with updated anatomical information in real-time [30]. 

Several augmented reality training models have been specifically developed for ESBS [31]. These training models allow trainees to gain exposure to a wide range of skull base pathologies before experiencing surgery with real-life patients. Synthetic tissue models, such as the UpSurgeOn system (UpSurgeOn SRL, Assago, Italy), incorporate augmented reality to provide anatomical exploration that is not otherwise possible with the unaided eye [32]. However, this technology is still in its infancy and requires thorough investigation. Advancements in augmented reality, and its growing integration into both neurosurgical training and practice, highlight the increasing importance of these neuronavigation-based technologies.

In ESBS, newer neuronavigation-compatible instruments have increased the utility of these systems. The electronics required for neuronavigation are incorporated in the instrument, which provides greater ergonomics and maneuverability for the surgeon compared to traditional instruments requiring external adaptors. Neuronavigation-compatible instruments also simplify the registration process. For example, malleable suction instruments can be bent during surgery without requiring re-registration. Other available instruments include drills and microdebriders.

However, neuronavigation has notable limitations. Optical image guidance is dependent upon camera line-of-sight and requires the patient to be firmly secured during surgery. Magnetic-based systems are prone to reduced accuracy from interference by external electromagnetic radiation or ferromagnetic instruments. Registration occurring on the face of the patient, as is typically carried out in ESBS, results in greater error at increased depths during surgery [23]. These imprecisions can be further exacerbated by brain shift. Re-registration at the site of interest is not without risk, with the potential for prolonged operative time and increased risk of complications, such as cerebrospinal fluid leak and impaired wound healing [33].

### 3.4. Robotics

Robotics have had limited use in skull base surgery relative to other surgical fields due to the anatomical constraints of the skull base and its narrow operative corridors. Developed primarily for general surgery, the larger footprint of robotic systems in the operating room, and the larger size of their instruments, has limited their endonasal use. Nevertheless, systems such as the da Vinci robot (Intuitive Surgical Inc., Sunnyvale, CA, USA), have been investigated in skull base surgery [34,35]. The incorporation of robotics has the potential to address the limitations of endoscopy—2D monocular vision, often requiring two co-surgeons—by providing 3D HD vision, requiring one surgeon, reducing the effects of physiological tremor, and increasing dexterity [36]. Robotic systems in conjunction with ESBS have been demonstrated to be feasible [37]. They continue to be investigated for improving access to skull base pathologies through combined approaches, such as the transoral (robotic)-transsphenoidal (endoscopic endonasal) approach. 

Robotic endoscope holders are also available, which can be adjusted either directly or with a joystick. New systems continue to be investigated preclinically and demonstrate significant progress over earlier models [38]. Notably, these systems offer articulation of the endoscope and improved ergonomics [39]. They can also reduce surgeon fatigue, improve concentration on tissue manipulation, and increase image stability without requiring a second surgeon [40]. The advantages of robotic systems have the potential to further improve the practice of ESBS and its outcomes for patients.

However, robotics in ESBS remain a relatively new development due to their large size relative to the skull base, lack of haptic feedback, and lack of integration with other visualization systems. These systems may also disrupt the surgical collaboration inherent to four-handed, two-surgeon approaches. Currently, the published literature is limited to preclinical studies. Increased research on the utility of these promising systems is required, especially in the clinical context [41].

### 3.5. Intraoperative Imaging

#### 3.5.1. Real-Time Fluorescence Agents

Fluorescence agents have been used in ESBS to aid in the visualization of blood vessels and tumors [42]. These agents work through a mechanism involving electron excitation through the absorption of higher-energy light and the subsequent emission of lower-energy light as the electron returns to its ground state. Fluorescence agents can demarcate tumors from healthy tissue to aid in greater tumor resection [43].

Indocyanine green (ICG) is a fluorophore that binds to plasma proteins in the intravascular space and has a very favorable safety profile [44]. Being contained within the intravascular space, ICG has been used to visualize and avoid damage to blood vessels during surgery. ICG has been also studied in a variety of tumors, most notably pituitary adenoma. Fluorescence intensity measured within one minute of ICG infusion can differentiate healthy tissue compared to the pituitary adenoma. Other techniques have also been developed, such as second-window ICG, where high-dose ICG infusion 24 h before surgery can concentrate fluorescence within the pituitary adenoma [45].

5-aminolevulinic acid (5-ALA) is a fluorophore acting through the porphyrin synthesis pathway that is approved for intraoperative visualization of high-grade glioma [46]. 5-ALA has been studied in ESBS for multiple conditions, though results suggest limited utility in specific tumor types [47]. This agent can enhance the detection of tumor tissue otherwise difficult to visualize due to its location near the optic canal. Compared to a microscope, the proximity of an endoscope to the tissue can improve fluorescent signal detection for deeply located pathologies. Future knowledge about the properties and lesion-specific pharmacokinetics of 5-ALA can improve its use in ESBS [48].

On Target Laboratories (OTL)-38 is a fluorophore that targets folate receptor alpha [49]. This agent has been promising in detecting folate receptor-overexpressing tumors. In one prospective study, OTL-38 intraoperatively demonstrated high sensitivity and specificity in detecting non-functioning pituitary adenomas [50]. Evaluation of tumor resection margins revealed improved resection with OTL-38 compared to unaided visual inspection. However, one major limitation of OTL-38 is the lack of known folate receptor expression levels preoperatively, which may reduce sensitivity and increase the occurrence of false negative results [51]. These agents, and additional fluorophores such as sodium fluorescein, continue to be researched to improve visualization and patient outcomes in ESBS.

#### 3.5.2. Ultrasonography

Ultrasonography is based on the reverse piezoelectric effect, where an electrical current causes vibration of a crystal lattice, producing high-frequency sound waves that are reflected to a transducer to recreate an image [52].

Ultrasonography has not yet gained wide adoption in ESBS. Ultrasound probes have been assessed in pituitary tumor resection, with limited ability to evaluate skull base anatomical structures [53]. The recent development of smaller-sized probes with improved resolution may increase the utility of this technique in ESBS. One retrospective study found increased extent of resection and fewer complications for patients who underwent ultrasound-guided pituitary adenoma resection compared to traditional surgery [54]. However, the published experience is limited, and prospective studies remain to be reported.

**Figure 7:**
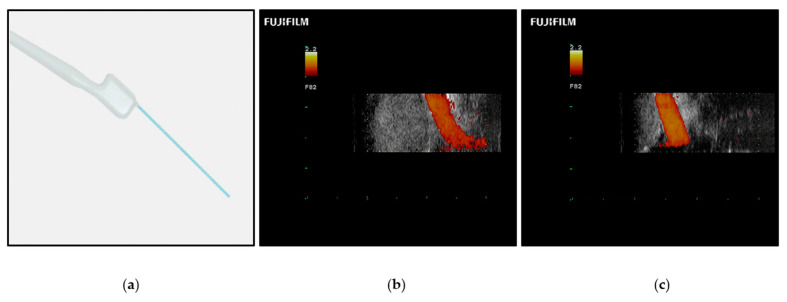
(**a**) Fujifilm pituitary guidance transducer (Fujifilm Healthcare Americas Corporation, Twinsburg, OH, USA). (**b**) Intraoperative Doppler ultrasonography showing a pituitary tumor and the cavernous segment of the internal carotid artery (ICA) before tumor debulking. (**c**) Intraoperative Doppler ultrasonography showing a pituitary tumor and the cavernous segment of the ICA after tumor resection. Reprinted/adapted with permission from Ref. [57]. 2022, Fujifilm Corporation.

Color Doppler ultrasonography, which labels fluid velocity with color, can identify vascular structures in the skull base, such as the internal carotid artery (Figure 7). New improvements in probe portability and resolution have led to the color Doppler microvascular probe. Compared to traditional Doppler probes, color Doppler microvascular probes have shown greater promise in identifying key vascular structures during ESBS [55]. However, image resolution and accurate structure identification are limited. The development of ultrasound contrast agents may further improve ultrasonography in ESBS. Contrast-enhanced ultrasonography has been reported for a variety of skull base pathologies [56]. This technique resulted in successful visualization of lesion tissue and high- and low- flow blood vessels compared to traditional ultrasonography. However, these contrast agents have not been tested using an endoscopic approach.

#### 3.5.3. Computed Tomography and Magnetic Resonance Imaging

Imaging modalities, such as computer tomography (CT) and magnetic resonance imaging (MRI), have enhanced the study and diagnosis of skull base conditions. CT uses X-rays and a mathematical process termed reconstruction to transform three-dimensional structures into two-dimensional cross-sectional images. MRI is based on nuclear magnetic resonance, primarily of hydrogen atoms, in an applied magnetic field that is detected and reconstructed into cross-sectional images without the use of ionizing radiation [58]. 

These imaging modalities have been used during surgery to assess skull base anatomy. Intraoperative CT and MRI have been used to evaluate residual tumor tissue, leading to improved extent of resection and progression-free survival [59]. Currently, intraoperative CT may be performed using mobile units, while intraoperative MRI requires designated imaging suites. One advantage of intraoperative imaging is the possibility of performing neuronavigation system re-registration during surgery, which is particularly useful in open cranial surgery where significant brain shift can occur. Re-registration provides updated imaging information to the surgeon and can identify intraoperative changes to anatomical structure [23]. However, intraoperative imaging has its limitations in ESBS. CT causes exposure to ionizing radiation, which limits its use in certain patient populations, including children and pregnant women [60]. MRI is time-consuming compared to other imaging modalities and can prolong surgery duration by up to 40 min [61].

### 3.6. Neuroanatomy

The evolution of ESBS has required revisiting known anatomical structures through the perspective of endoscopic endonasal approaches [62]. New endoscopic approaches have invited a fundamental reevaluation of skull base anatomy and established procedures. Improved anatomical knowledge has informed surgical techniques and made endoscopic approaches a first-line choice for certain conditions, such as craniopharyngiomas [63]. Detailed descriptions of the microsurgical anatomy of the skull base, and the common sites of its pathologies, have enabled surgeons to appropriately plan and perform ESBS.

**Figure 8:**
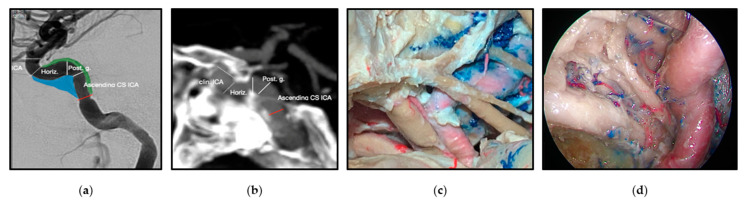
The cavernous sinus spaces and the cavernous segment of the internal carotid artery (ICA) from imaging, microsurgical transcranial and endoscopic endonasal perspectives. (**a**) Angiogram, lateral view of the ICA. (**b**) CT angiogram, lateral view of the ICA. The relationship of the ICA with the sphenoid bone is observed. (**c**) Cadaveric dissection demonstrating a superolateral view of the middle fossa and ICA via a transcranial approach. (**d**) Cadaveric dissection demonstrating an endoscopic endonasal view of the relationship of the cavernous ICA with the nerves on the lateral wall of the cavernous sinus. Reprinted/adapted with permission from Ref. [18]. 2022, Erion Junior de Andrade, M.D., M.Sc.

One example of the reevaluation of the microsurgical neuroanatomy is the cavernous sinus (Figure 8). The division of the cavernous sinus and middle fossa into triangles has been traditionally used for transcranial open approaches, however, this technique is not adequate for endoscopic endonasal approaches. Recently, a 360-degree division of potential spaces of tumor extension was described to provide anatomical guidance for surgical approach selection [62].

Detailed descriptions of the skull base are especially important for areas with complex anatomy and multiple available approaches. The anterior, middle, and posterior cranial fossa can be accessed from the front of the skull near the eye (transorbital), from the sides of the skull base (transpterygoid), from the back of the skull base (transcondylar), via the region near the pituitary gland (parasellar), and near where the spinal cord exits the cranium (clival and petroclival) [62,63,64,65,66,67,68,69]. Knowledge of neuroanatomy continues to grow with the development of expanded endoscopic approaches that encompass greater areas of the skull base [70]. The increasing application of endoscopic approaches in pediatric surgery has further refined the current understanding of skull base development [71]. The knowledge of surgical micro-neuroanatomy should be used in combination with available technology as the mainstay of any surgical procedure.

### 3.7. The Exoscope

**Figure 9:**
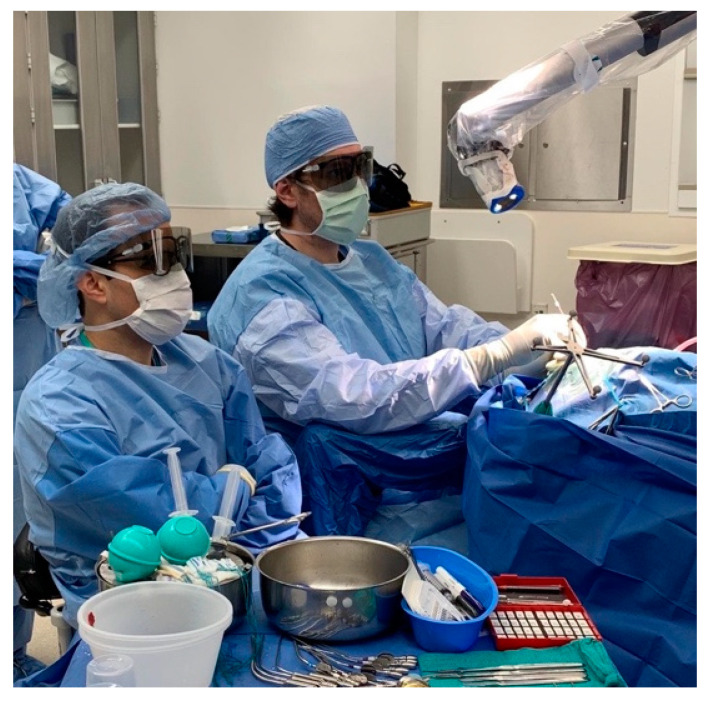
The surgical exoscope in use during a neuro-surgical procedure. Reprinted/adapted with permission from Ref. [12]. 2022, Pablo F. Recinos, M.D.

In addition to the endoscope, work has been performed to develop the exoscope: a telescopic intraoperative visualization device with HD video resolution [72] (Figure 9, Video S1). Unlike the endoscope, the exoscope is positioned outside the body of the patient. It provides greater magnification, generates a wider focal distance, incorporates image enhancement, and provides 3D visualization. These features can improve anatomical visualization, increase surgeon comfort with better ergonomics, and facilitate teaching [73].

Exoscopy is a relatively new development in skull base surgery and further study is required to understand its strengths and limitations. Identified limitations include tissue differentiation, specifically bleeding tissue, the loss of stereoscopy, and the need for improved integration with existing technologies, such as fluorescence agents and endoscopy [74]. Endoscopes used with exoscopes have been reported to improve the visualization of operative blind spots. The exo-endoscopic approach can provide not only a wider field of view, but also better instrument positioning and improved viewing perspective for shared observation by operating room personnel [75]. Continued operative experience with the exo-endoscopic approach can further advance the complementary use of these technologies in ESBS [76].

## 4. Future Innovations

The current state of visualization in ESBS demonstrates significant technological progress over time. Exciting advancements await following the refinement of existing technologies and the development of novel ones. Improved light sources with lower energy utilization and heat generation can limit thermal tissue damage. A reduced risk of tissue damage would permit increased illuminance and/or a shorter working distance, which would increase image brightness and resolution to improve surgical precision. Image resolution can be further enhanced with improved light sensors incorporated into endoscopes. Three-dimensional endoscopy can redefine the operative experience by more accurately recreating skull base anatomy for the surgeon [77].

Fluorescence agent guidance in ESBS represents a promising avenue for improving tumor extent of resection. However, the current published literature is largely limited to descriptive studies, such as case series. A greater number of studies with large sample sizes and standardized protocols would increase the level of evidence evaluating the strengths and limitations of fluorescence agents in ESBS [78]. Multi-institutional collaboration can significantly support this goal by facilitating large-sample trials. Research on the concurrent use of multiple highly sensitive and specific fluorophores (e.g., one targeting a specific brain tumor and one identifying blood vessels) may address the limitations of single-fluorophore protocols. Continued advances may lead to streamlined endoscopes that can concurrently detect multiple fluorophores in real-time. Beyond fluorescence, refined contrast agents for contrast-enhanced ultrasonography microprobes can improve endoscopic identification of critical vascular structures to reduce complications [79]. Prospective, large-sample trials would increase the level of evidence assessing the utility of novel contrast agents in ESBS.

Improved spatial resolution and image acquisition time for intraoperative CT or MRI can provide more imaging data and address brain shift for neuronavigation systems [80]. The site-specific registration at the site of resection and streamlined intraoperative re-registration can enhance neuronavigation accuracy [81]. Augmented reality neuronavigation represents a novel avenue for providing accurate knowledge of patient-specific anatomy. Advancements in the incorporation of intraoperative imaging can facilitate real-time updated augmented reality, which would be especially useful for eloquent brain regions. Improved image processing and data storage can attenuate the computational demands of these computer-generated methods [82]. Beyond the operating room, these systems can provide trainees with simulation opportunities and additional anatomical guidance during surgery. Increasing trainee access to cadaveric dissections through dedicated dissection laboratories and published atlases can expand the collective knowledge of skull base anatomy. The refinement of new technologies, such as the exoscope, and their incorporation with the endoscope can further improve field visualization to promote safe ESBS for patients [83].

## 5. Conclusions

Skull base surgery has experienced significant progress in surgical technology (e.g., reconstruction materials, light sources, microscopy, endoscopy) over the past two centuries. These advances have been facilitated by improved visualization techniques, enabling modern ESBS. Current state-of-the-art endoscopy uses ultra-HD/4K image resolution and 3D vision. Fluorescence agents such as ICG, 5-ALA, and OTL-38 have shown initial success in identifying residual tumor tissue to improve extent of resection. Contrast-enhanced ultrasonography has the potential to help identify important vascular structures; however, there is a need to evaluate this technology in ESBS in large-sample studies with standardized protocols. Neuronavigation systems today offer electromagnetic tracking, which includes specialized instruments that are more maneuverable and are not limited by line-of-sight, unlike optical image guidance. Advancements in virtual reality and augmented reality provide surgeons with more patient-specific anatomical information when planning and performing surgery. These systems have begun to shape surgical education by providing trainees with part-task training, and their exact role in education remains to be defined. The exoscope serves as an additional resource for enhancing intraoperative visualization with a wider focal distance, 3D vision, and improved instrument positioning.

These visualization techniques have increased the use and safety of modern ESBS. With numerous existing and developing tools, sometimes with overlapping capabilities, the skull base surgeon must have a deep understanding of the strengths and limitations of each visualization technique, and their judicious use can continue to improve patient outcomes.

## Data Availability

Not applicable.

## Supplementary Materials

The following supporting information can be downloaded at: https://www.mdpi.com/article/10.3390/brainsci12101337/s1, Video S1: Exoscopic Supraorbital Approach for Resection of a Tuberculum Sellae Meningioma.
